# A Multiwell-Based Detection Platform with Integrated PDMS Concentrators for Rapid Multiplexed Enzymatic Assays

**DOI:** 10.1038/s41598-018-29065-7

**Published:** 2018-07-17

**Authors:** Xi Wei, Vu Q. Do, Sang V. Pham, Diogo Martins, Yong-Ak Song

**Affiliations:** 1 Division of Engineering, New York University Abu Dhabi, Abu Dhabi, United Arab Emirates; 2 Department of Chemical and Biomolecular Engineering, Tandon School of Engineering, New York University, Brooklyn, United States; 3 School of Transportation Engineering, Hanoi University of Science and Technology, No1 DaiCoViet, Hanoi, Vietnam; 4 NOVA Medical School, Faculdade de Ciências Médicas, Universidade Nova de Lisboa, Lisboa, Portugal

## Abstract

We report an integrated system for accelerating assays with concentrators in a standard 12-well plate (ISAAC-12) and demonstrate its versatility for rapid detection of matrix metalloproteinase (MMP)-9 expression in the cell culture supernatant of breast cancer cell line MDA-MB-231 by accelerating the enzymatic reaction and end-point signal intensity via electrokinetic preconcentration. Using direct printing of a conductive ion-permselective polymer on a polydimethylsiloxane (PDMS) channel, the new microfluidic concentrator chip can be built without modifying the underlying substrate. Through this decoupling fabrication strategy, our microfluidic concentrator chip can easily be integrated with a standard multiwell plate, the *de facto* laboratory standard platform for high-throughput assays, simply by reversible bonding on the bottom of each well. It increases the reaction rate of enzymatic assays by concentrating the enzyme and the reaction product inside each well simultaneously for rapid multiplexed detection.

## Introduction

To run molecular bioassays in a highly parallel fashion, the *de facto* laboratory standard platform is a microplate containing multiple wells, of which the number can vary anywhere from 6 to 1536. To run an assay in the wells, the standard method is to mix the analyte of interest with an appropriate reactive reagent and then dispense it into each well for static incubation at optimum temperature. Depending on the assay type used, the amount of reaction product, for example, a fluorescent molecule, can then be measured with a spectrofluorometer, such as a plate reader or charged-coupled device (CCD). Alternatively, the target molecule can be captured by the molecular binding probes immobilized on a plate well. The binding event can then be detected in a sandwich assay by adding a second binding probe with a fluorescent tag.

Several microfluidic devices based on the nonlinear electrokinetic phenomenon - ion concentration polarization (ICP) have been developed to preconcentrate biomolecular samples. Micro-/nanochannel interfaces^1–5^, ion-permselective membranes^6–11^ or embedding bipolar electrodes^12–14^ have been adopted in device design to optimize the preconcentration performance and increase the reaction rate and detection sensitivity of immunoassays^1,15–18^ as well as of those of standard enzymatic assays^19–21^. Lee *et al*. reported concentration enhanced cell kinase assay in a micro-/nanofluidic platform directly from cell lysates and demonstrated a 25-fold increase in reaction rate and 65-fold enhancement in sensitivity^19^. The formation of a highly concentrated biomolecular plug results from the ion permselectivity of nanoporous membrane/channels in combination with an electroosmotic flow that transports biomolecules from the anodic reservoir towards the ion depletion zone near the membrane and increases its concentration via electrokinetic trapping (ET)^4,22,23^. There have also been attempts to multiplex the electrokinetic concentrators by patterning a long Nafion strip on a glass slide and plasma bonding an array of 120 microchannels in a polydimethylsiloxane (PDMS) chip^6^. Despite Nafion being the common choice of material for cation-selective membrane^6,15,17–21,24–29^, the microflow patterning technique used to pattern Nafion resin on a glass substrate, although simple in principle, has the following limitations: it is not compatible with cleanroom microfabrication technique; it leads to variable membrane thicknesses; and it allows for “hidden” nanochannels along the membrane edges due to an imperfect PDMS sealing that acts as leakage holes^29^ and dominate the ionic transport^30^. These imperfections lead to the compromise of the ionic permselectivity of the membrane. Above all, this microflow patterning method limits the integration flexibility of the electrokinetic concentrator device on uneven or spatially confined surfaces such as wells of a microplate. To bypass such limitations, a PDMS preconcentrator with different fabrication method and ion-selective polymer has been developed in our group^31,32^. Poly(3,4-ethylenedioxythiophene)-poly(styrene sulfonate) (PEDOT:PSS) dispersion in H_2_O has low viscosity (10–30 cP at 20 °C) and consequently can be directly deposited on a very confined area of a glass or PDMS substrate using a piezoelectrically-actuated micropipette. If the conductive polymer is directly printed on a PDMS channel surface, the fabrication of a PDMS concentrator chip can be entirely decoupled from the substrate where the capture probes for detection are usually surface-immobilized. This decoupling fabrication strategy requires no additional substrate surface modification and reduces complexity in the alignment of the concentrator device to the ion-permselective membrane deposited on a flat substrate. In addition, spreading of the PEDOT:PSS solution can be controlled by printing it on a predefined pattern of a PDMS chip to achieve more uniformity in the size of ion-permselective membrane layer^31^. This approach further reduces the risk of compromising the stability of the preconcentration performance due to an incomplete sealing between the PEDOT:PSS membrane and the PDMS chip. Finally, this new decoupling fabrication strategy allows the integration of a PDMS preconcentrator chip to any confined and uneven surfaces such as the bottom surface of wells in a microplate.

**Figure 1:**
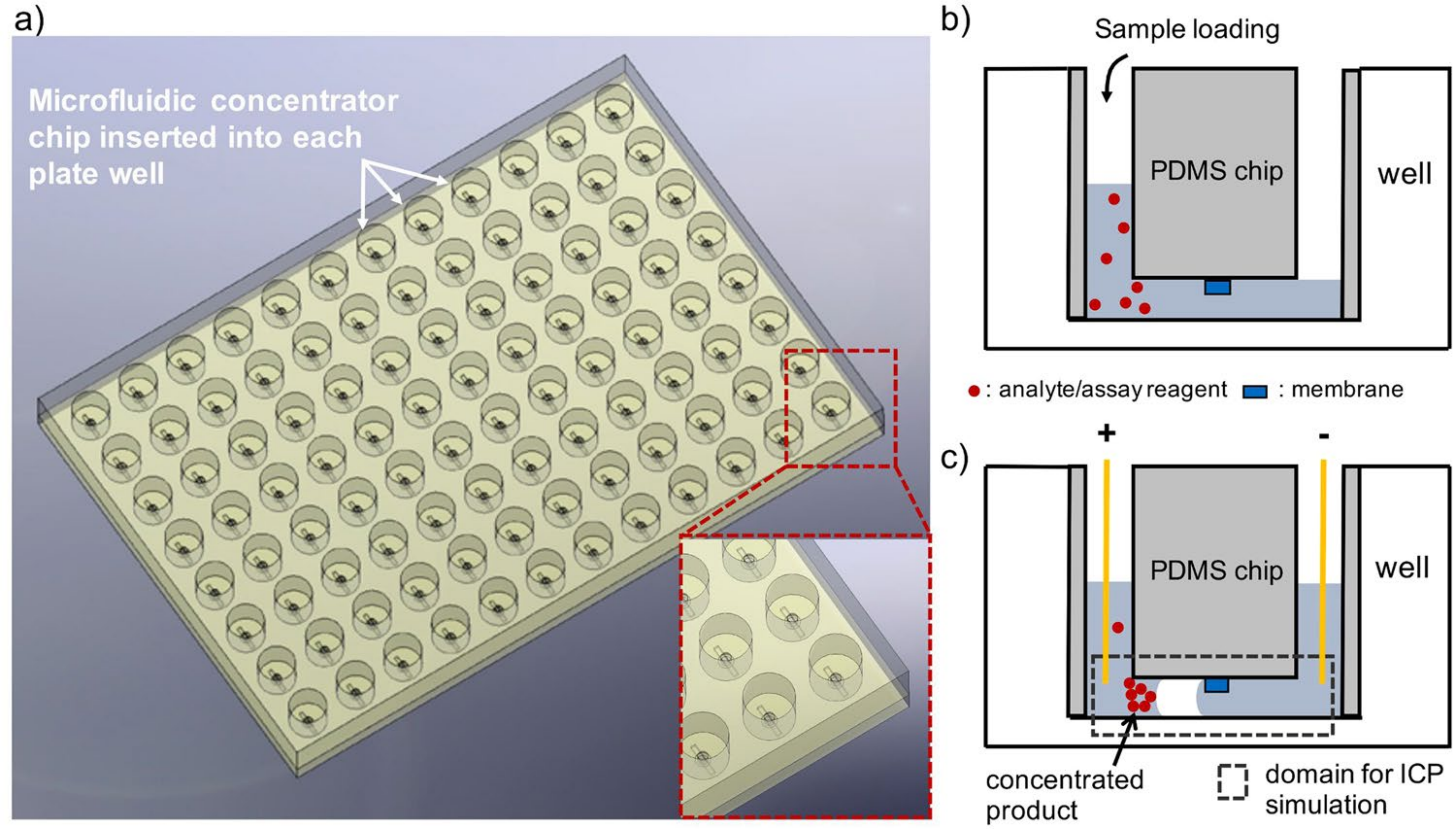
The working principle of a rapid detection in the integrated detection system ISAAC by accelerating the assay with a PDMS concentrator integrated into each well. (**a**) Schematic of a standard microplate-based ISAAC with PDMS concentrator chips inserted into the wells and reversibly bonded onto the bottom surfaces. (**b**) A reaction mixture containing an analyte (e.g., MMP-9) is loaded into the concentrator chip. (**c**) A molecular concentration plug containing the reaction mixture as well as the reaction product forms on the anodic side of the PEDOT:PSS membrane as a result of the balance between the ion depletion zone and electroosmosis when voltage is applied across the microchannel. The dotted area indicates the computational domain for numerical simulation of ICP.

In this work, we propose an **I**ntegrated **S**ystem for **A**ccelerating **A**ssays with **C**oncentrators (**ISAAC**) following the standard microwell plate layout and demonstrate its versatility in the rapid matrix metalloproteinase (MMP)-9 detection from breast cancer cell line MDA-MB-231 by accelerated enzymatic assay. The primary motivation of this work was to demonstrate the integration capability of our microfluidic preconcentrator chip in PDMS to a standard laboratory assay platform such as a multiwell plate so that multiplexed assays can readily be performed in general laboratory settings with minimal operational and instrumental changes as well as training requirements. Such an effort could lead to higher acceptance of microfluidic devices for analysis and diagnosis in research and clinical laboratories. In our view, this standard assay platform-adapted approach seems more viable than the other way around in which laboratories reformat their standard assay platform and adapt themselves to the microfluidic platform. The proposed detection platform was assembled by reversibly bonding PDMS concentrator chips onto the bottom surfaces of wells in a standard microplate (Fig. 1a). Each concentrator chip was then loaded with a reaction mix (Fig. 1b), and the reaction rate of the bioassay was enhanced by concentrating the enzyme sample and the reaction product inside each concentrator chip. The electrode array, which was mounted on a printed circuit board (PCB), was used to provide external voltage to each microfluidic chip to initiate electrokinetic preconcentration based on ICP inside each microchannel simultaneously with a single-step operation (Fig. 1c). As a result, the enzyme and the substrate together with the fluorescent reaction product were concentrated leading to reduced reaction time and amplified end-point signal intensity. Since the enzymatic reaction was running inside a microfluidic channel, the required sample volume was significantly lower than in a standard multiwell plate without electrokinetic preconcentrator (≤ 40 μL). The technical innovation of our work lies in the simple and straightforward interfacing of microfluidic concentrator chips with a standard microplate-based assay platform to enable highly multiplexed and rapid detection of biological analytes at high sensitivity. This integration effort could ultimately lower the entry barrier for microfluidic devices into laboratory settings.

## Results and Discussion

### Numerical Simulation of ICP

**Figure 2:**
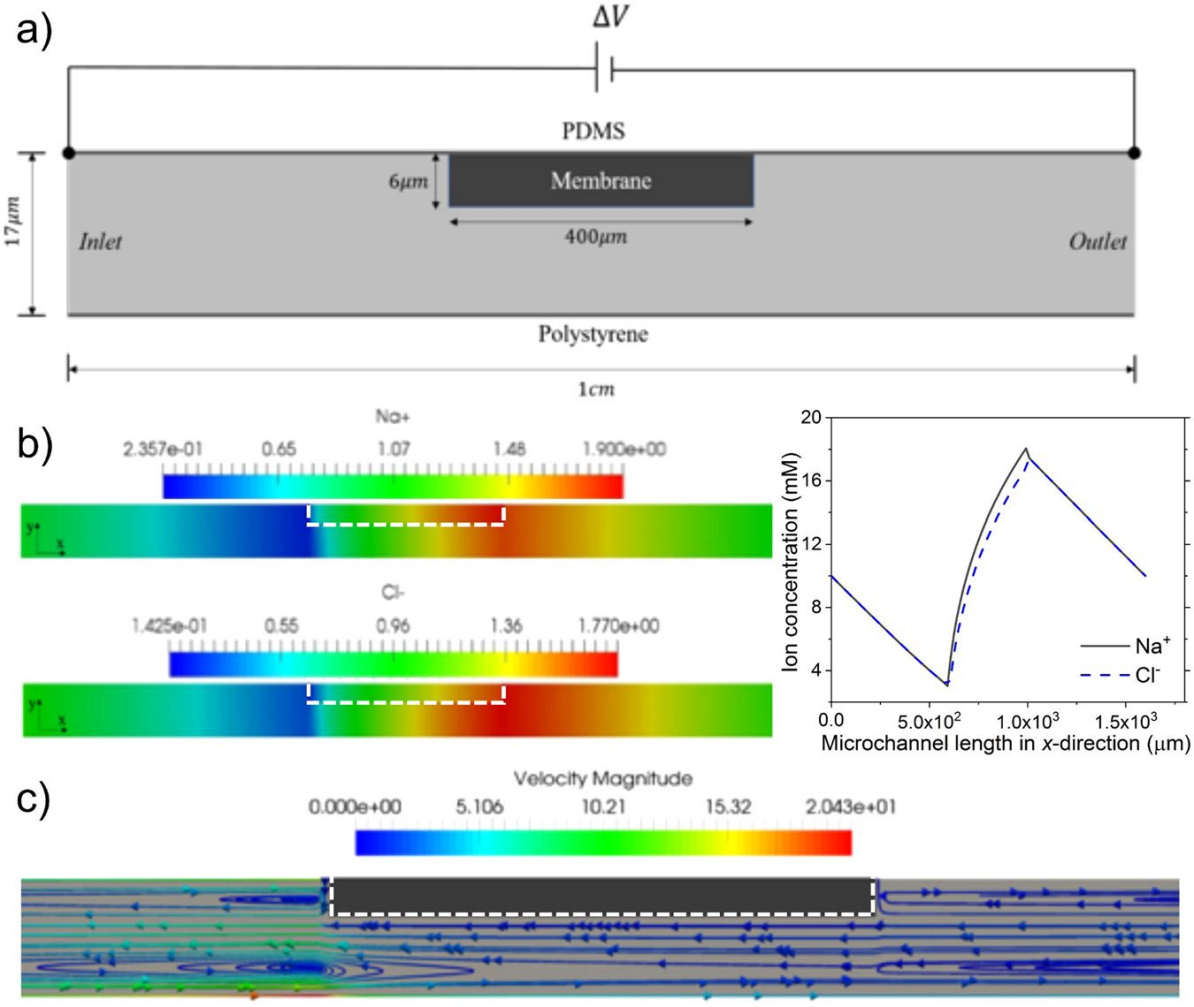
Simulation results of ICP in the microchannel with 50 V at steady state. (**a**) Computation domain of 400 μm × 6 μm ion-permselective membrane and 1 cm × 17 μm microchannel for ICP simulation at steady state. (**b**) Due to the ionpermselectivity of the membrane, an ion depletion region forms on the anodic side of the membrane, while an ion enrichment region forms on the opposite side of the membrane. The graph depicts ion concentration distributions of Na^+^ and Cl^-^ ions along the horizontal line of membrane surface (at microchannel height *y* = 11 μm). (**c**) The flow profile in the microchannel shows vortices formed in the channel due to ICP in the microchannel. The velocity is scaled by 31.3 μm/s. The membrane location is outlined by the dotted line in white.

The simulation results for ICP inside a microchannel with an ion-selective membrane printed on the top of the channel are shown in Fig. 2. The computational domain for this simulation is indicated with a dotted box in Fig. 1c. Even though the ion-selective membrane with a thickness of 6 μm is only partially covering the microchannel with a total height of 17 μm (Fig. 2a), an ion depletion zone forms on the anodic side (left) and an ion enrichment zone on the cathodic side (right) of the membrane, as shown in Fig. 2b. As the membrane transports only cations with larger transport number than electrolyte, the concentration of Na^+^ ions in the region next to the membrane on the left becomes depleted from an initial concentration of 10 mM to a concentration of ~2mM. To maintain electroneutrality, Cl^-^ ions are also depleted in the same region, while on another side of the membrane, Cl^-^ ions accumulate and form an enrichment region due to the ion selectivity of the membrane which is not able to conduct them through the membrane (Fig. 2b). Concentrations of both ions vary linearly from the depleted region to the inlet bulk (Fig. 2b), which is in agreement with previous studies^33,34^.

Due to electroosmosis on PDMS and polystyrene surfaces, fluid flow is driven from left to right, along the channel. The membrane blocks the flow and results in a back-flow vortex on the PDMS surface, as can be seen in Fig. 2c. In the current case, there is no electroconvective flow initiated by flow in the boundary layer of the membrane^33,34^, as the depleted concentration is not low enough to trigger instability of the boundary layer. At higher applied voltage, as the depleted concentration is decreased to the level of developing extended space charge layer, the electroconvective flow is expected to occur characterized by strong vortical flow on the membrane surface^33–35^. Target biomolecules carried by the electroosmotic flow as well as the ions in the electrolyte are eventually accumulated in front of the depletion zone (anodic side) and continue concentrating under the balance between electroosmotic flow and repulsion from the depletion zone.

### Device fabrication and assembly

**Figure 3:**
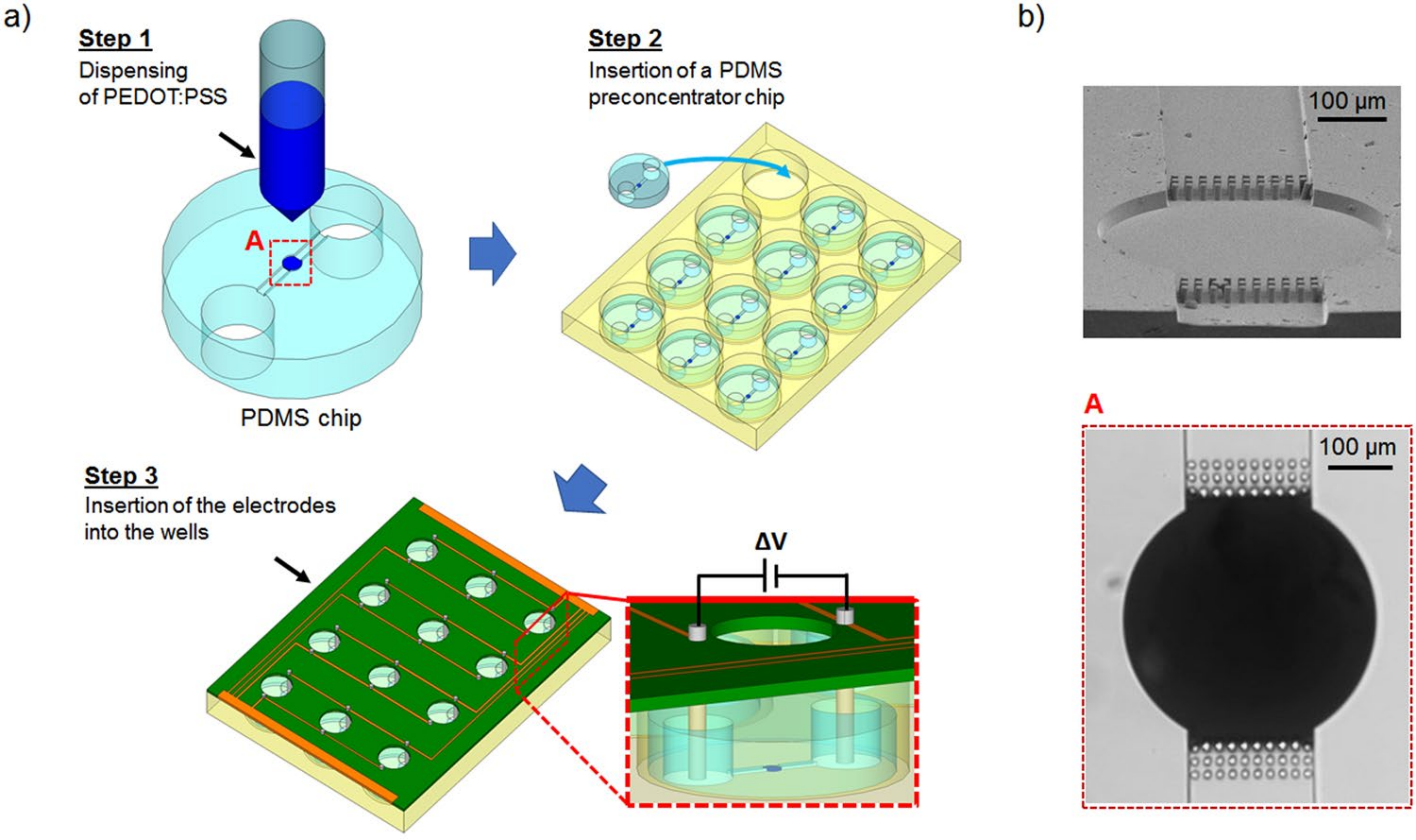
Schematic of ISAAC-12 fabrication process. (**a**) Fabrication of a concentrator by printing PEDOT:PSS directly onto the top of a PDMS channel. (**b**) Improved PEDOT:PSS membrane printing on PDMS chip with additional micropillar structures on both sides of the central circular pattern by using layer by layer printing technique. Upper: SEM image of the micropillar structure. Each micropillar was 14 μm in height and 10 μm in diameter. The space between each micropillar was 10 μm; lower: an optical image of a ~6 μm thick membrane on PDMS chip after printing.

The fabrication process of ISAAC following a standard 12-well plate layout (ISAAC-12) is illustrated in Fig. 3a. In previous studies^32,36^, PEDOT:PSS was printed on the glass substrate followed by PDMS reversible bonding. These studies indicated that increasing membrane thickness or using higher conductivity grade PEDOT:PSS can both improve membrane performance. However, the repeatability of device fabrication was limited by the membrane printing step. The device yield was only ~34% when depositing a ~6 μm thick membrane on the glass substrate by using the layer-by-layer printing technique^32^. The common device failure was mainly due to the irregular shape of the membrane that resulted from the mismatch between two membrane layers in diameter because of the different surface conditions for the first and second layer (Fig. S1 in Supplementary Information). The new printing strategy of depositing PEDOT:PSS directly onto the PDMS channel instead of the glass substrate (Fig. 3a, step1) mitigated this fabrication issue. Besides the circular pattern on the PDMS chip which confined an overflow of PEDOT:PSS solution, an additional micropillar array (3 rows of 10 micropillars, 14 μm in height, 10 μm in diameter) on both sides of the circular pattern also supported to increase the thickness in a single dispensing step as well as the shape of the membrane layer by blocking the flow of the PEDOT:PSS solution into the side channels due to the surface tension between the micropillars and PEDOT:PSS solution.

**Figure 4:**
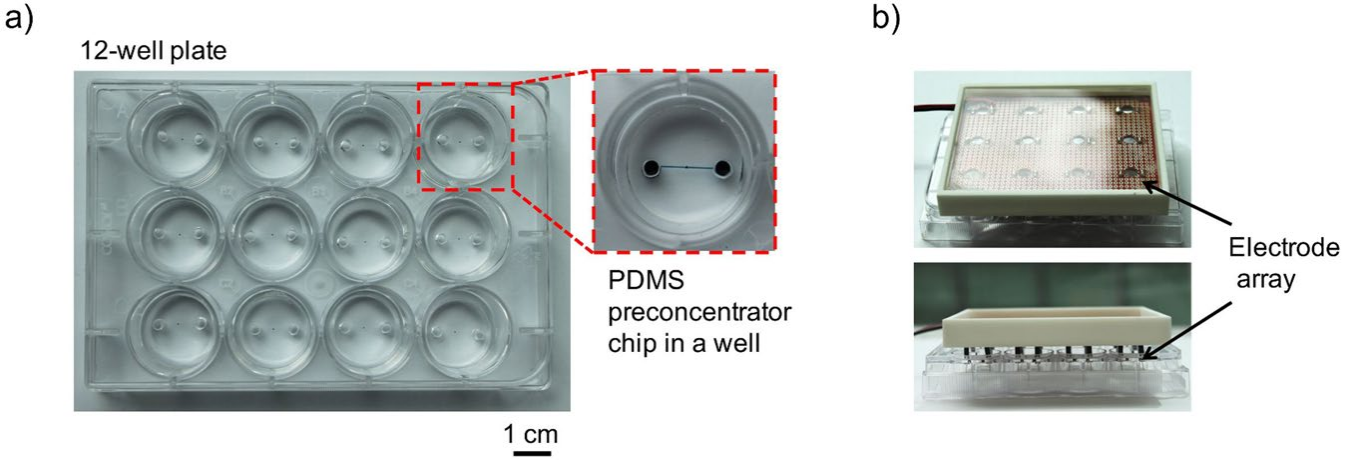
A photograph of the ISAAC-12 platform based on a 12-well plate. The ISAAC device comprises three parts: a 12-well plate, 12 PDMS concentrator chips (one is shown in the inset) and an electrode array. (**a**) 12-well plate after inserting PDMS concentrator chips. (**b**) ISAAC assembled with an electrode array for operation. Upper: top view of ISSAC-12; lower: side view of ISSAC-12.

Once placed into the wells and reversibly sealed against the bottom surfaces (Fig. 3a, step 2), the electrodes mounted on the PCB were inserted into the corresponding chip reservoirs through which the reaction mix was loaded, as shown in Fig. 3a, step 3). Figure 3b shows the micropillar array before and after printing of PEDOT:PSS solution in the microchannel. With this new fabrication method, the device yield significantly improved to 83% with a PEDOT:PSS membrane thickness of 5.9 ± 1.2 μm. The fluorescence signal released from the enzymatic assay was concentrated and recorded through the observation window on the PCB board. The final assembly of the ISSAC-12 platform, as shown in Fig. 4, required less than 5 min. We believe that this facile integration method could allow increasing the number of the concentrators even to a higher number, 48 or potentially 96, for high-throughput analysis without adding significant complexities.

### Characterization of ISAAC-12 performance with DNA

To compare the concentration performance of ISAAC-12 with our previous microfluidic concentrators, we first measured the preconcentration factor (PCF) for DNA as the test analyte. ISAAC device could preconcentrate DNA samples of various initial concentrations from 0.1, 1 and 10 nM with a PCF of 1134 ± 243 (Fig. S2 in Supplementary Information). This PCF was approximately one order of magnitude higher than that of the previous device with PEDOT:PSS printed on the PDMS channel and bonded to a glass substrate^31^. This increase in PCF can be explained by the increased thickness of PEDOT:PSS membrane in ISAAC, twice as thick as it was before (~3 μm), and thus, higher surface conductance and lower inversed Dukhin number^6^. However, compared to other devices, in which a similar thickness of PEDOT:PSS layer at ~6 μm was printed on the glass substrate^32^, PCF of ISAAC was more than 10-times lower due to the lower surface zeta potential value of polystyrene compared to that of glass. Under a low ionic strength of 10 mM and neutral pH, the zeta potentials of both polystyrene (~ −60 mV) and PDMS (~ −40 mV) surfaces are lower than that of a glass substrate (~ −100 mV)^37,38^. The lower zeta potential led to lower electroosmotic velocity inside the microchannel of ISAAC and consequently to a slower transport of biomolecules towards the ion depletion zone.

### Accelerated MMP-9 detection in ISAAC-12

The reaction rate of enzymatic assays is governed by the Michaelis-Menten Equation^39^ as follows:

1 $$v={v}_{max}\frac{{c}_{substrate}}{{k}_{m}+{c}_{sub{\rm{s}}trate}}={k}_{cat}{c}_{enzyme}\frac{{c}_{substrate}}{{k}_{m}+{c}_{substrate}},$$

where $v$ is the reaction rate, ${v}_{max}$ is the maximum reaction rate, ${c}_{substrate}$ is the concentration of a substrate, and ${k}_{m}$ is the Michaelis constant, which is defined as the substrate concentration at half of the maximum velocity. ${k}_{cat}$ is the catalytic constant of an enzyme, and ${c}_{enzyme}$ is the total enzyme concentration.

**Figure 5:**
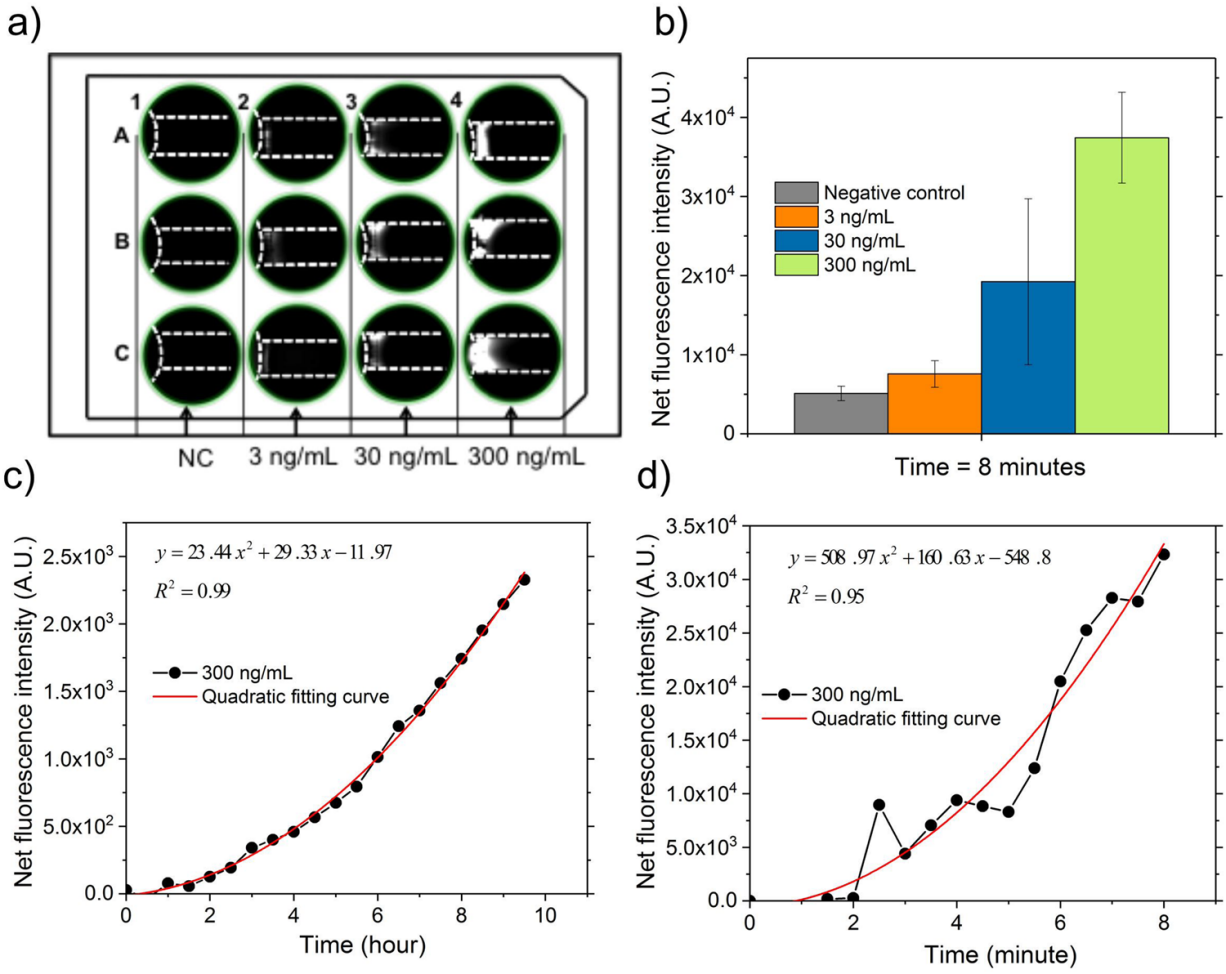
Characterization of the accelerated detection of MMP-9 with an initial concentration of 0, 3, 30 and 300 ng/mL in ISAAC-12. (**a**) Fluorescence images of concentrated 5-FAM in microchannels after 6 min. indicating the detection of MMP-9 adjacent to the membrane. Columns 1–4 for MMP-9 concentration 0–300 ng/mL with triplicates for each concentration along rows A–C. NC: negative control. (**b**) Measurement of the net fluorescence intensity of 5-FAM after 8 min. of the experiment: a significant fluorescence signal intensity increase with MMP-9 level from 0 to 300 ng/m. (**c**) Evaluation of MMP-9 reaction speed in a microfluidic channel under standard incubation without ICP as reference and (**d**) ISAAC-12 based on quadratic fitting curve. Purified MMP-9 sample was 300 ng/mL and normalized against negative control. The derivative (*y*′ = 2*ax* + *b*) of quadratic equation (*y* = *ax*^2^ + *bx* + *c*) was used to estimate the reaction rate.

At low ${c}_{enzyme}$ or high$\,{c}_{substrate}$, ${v}_{max}$ is directly proportional to ${c}_{enzyme}$, which was the case in this study with low abundance of MMP-9 and saturated FRET peptide. At steady state without any electrokinetic preconcentration, all the active sites of MMP-9 were occupied and as a result, the reaction rate was limited. In each microchannel of ISAAC, however, the increase of MMP-9 concentration adjacent to the PEDOT:PSS membrane resulted in higher reaction rate according to eqn. (1). Since the product of the enzymatic reaction, 5-FAM, was concentrated simultaneously, the detection sensitivity was further improved by the amplified fluorescence signal intensity of 5-FAM. At a constant DC voltage of 10 V, fluorescence signal intensity of 5-FAM from the reaction mix with an initial concentration of standard MMP-9 at 0, 3, 30 and 300 ng/mL in column 1, 2, 3 and 4, respectively, was recorded to monitor the reaction progress, as shown in Fig. 5a. The intra-assay and inter-assay coefficient of variability was 9.80% and 9.45%, respectively (Table S1 in Supplementary Information). Due to water evaporation during printing step, the viscosity change of PEDOT:PSS solution caused a variation of membrane thickness, as discussed under “Device fabrication and assembly”, that led to the variation in signal intensity among the triplicates for each concentration, as well as for each device. The assay coefficient of variability could be further improved through printing under humidity or temperature control.

The reaction signals resulted from the samples with various MMP-9 initial concentrations were monitored by mapping the net increase of fluorescence signal intensity in each well and quantified after 8 min. of electrokinetic preconcentration, as shown in Fig. 5b. The signal increase in negative control (NC) sample was due to free 5-FAM present in the background reaction reagent, which was possibly not completely removed by purification steps during FRET synthesis process. After 5 min., there was no further signal increase in NC sample observed, indicating that there was no additional 5-FAM released from the presence of MMP-9. However, in the samples with MMP-9 at 3, 30 and 300 ng/mL, the fluorescence signal intensity continued to increase over time, as well as the signal intensity differences between different concentrations. After 8 min, a signal increase of 1.5-, 3.8- and 7.3-fold was measured in those samples with MMP-9 at 3, 30 and 300 ng/mL compared to the NC samples, respectively. As a comparison, it took over ~10 hours of static incubation to measure a detectable level of fluorescence signal at 300 ng/mL of MMP-9 in the microfluidic channel (1.5-fold increase compared to NC sample), or in the 384-well plate with an optical readout using a plate reader (3.6-fold increase compared to NC sample) (Fig. S3 in Supplementary Information). Based on the quadratic fitting curve, the reaction rate of 300 ng/mL MMP-9 by standard incubation method in the microchannel was calculated as $46.88x+29.33$ ($x$ − reaction time; unit: hour) (Fig. 5c), while in ISAAC-12 it was estimated to be $1017.94x+160.63$ ($x$ – reaction time; unit: minute) (Fig. 5d). In fact, after 8 min. in ISAAC-12, the reaction rate reached 8304.15 RFU (relative fluorescence unit)/min, more than a 10^3^-fold increase compared to 404.37 RFU/h (= 6.7395 RFU/min) after 8 h of standard incubation in the microchannel.

**Figure 6:**
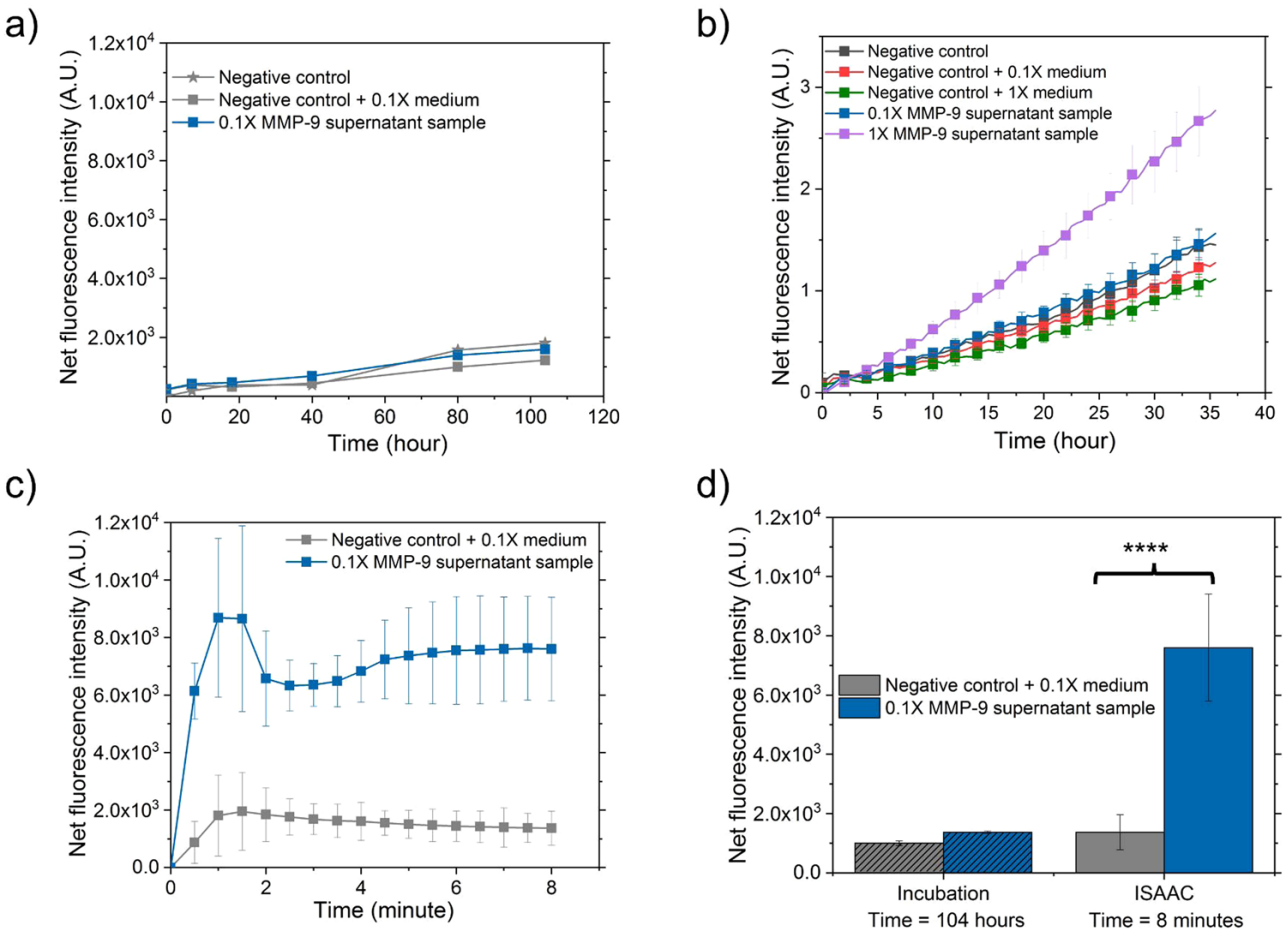
Rapid detection of MMP-9 from 10-fold diluted MDA-MB-231 breast cancer cell culture supernatant in ISAAC-12. (**a**) The reference study of MMP-9 assay with culture supernatant samples including one positive 0.1X MMP-9 supernatant sample and two negative controls (one with 0.1X fresh medium, one with assay buffer) in the microchannels incubated over 104 hours. The fluorescence signal intensity of 0.1X MMP-9 supernatant sample was below the detection limit. (**b**) A 36-hour incubation study in the 384-well plate of MMP-9 assay with two positive samples from culture supernatant samples including 1X MMP-9 from culture supernatant and its 0.1X dilution, and three negative control samples (two with fresh medium and 0.1X medium respectively, one with assay buffer). Only 1X MMP-9 from culture supernatant sample showed 2.5-fold signal intensity increase compared to its negative control sample. 0.1X MMP-9 from culture supernatant sample could not be detected since no significantly higher signal intensity level was observed compared to the negative control (p = 0.12 at significance level α = 0.05, Kruskal-Wallis ANOVA test). (**c**) The signal intensity of 0.1X MMP-9 in ISSAC-12 was clearly detectable compared to the signal intensity of its negative control sample over 8 min. experiment. (**d**) The signal intensity increase in 0.1X MMP-9 sample from culture supernatant to its negative control by standard incubation and ISAAC. The signal intensity levels were significantly different when the enzymatic reaction was performed in ISAAC (p < 0.0001).

The detection of MMP-9 directly from MDA-MB-231 cell culture supernatant further validated the performance of ISAAC-12 in accelerating the enzymatic reaction. Benchmarking study in the microchannel without ICP-based electrokinetic preconcentration showed no significant difference (p = 0.75) between positive sample 0.1X MMP-9 supernatant sample (10X dilution of the culture supernatant) and other negative control samples after 104 hours of standard incubation (Fig. 6a). A 36-hour study in the 384-well plate also confirmed that the 0.1X MMP-9 supernatant sample was not detectable since its signal intensity was below the detection limit of the plate reader, while the signal intensity of the 1X MMP-9 supernatant sample (the undiluted supernatant) was increased by ~2.5-fold in comparison with its negative control (Fig. 6b). As a comparison, a 4.4-fold signal intensity increase from the 0.1X MMP-9 supernatant sample was measured within 8 min. of ICP-accelerated assay in ISAAC (Fig. 6c and d). The signal intensity level was between the reference signal intensity levels of 3 ng/mL and 30 ng/mL (data obtained from Fig. 5b), indicating that the 0.1X MMP-9 concentration from cell culture supernatant was in the range of 3 to 30 ng/mL. From the standard calibration curve based on the microwell-incubation method, the concentration of the 0.1X MMP-9 supernatant sample was estimated to be at ~16 ng/mL (Fig. S4 in Supplementary Information), which was in good agreement from the evaluation by ISAAC-12. Noticeably, the signal intensity of the negative control sample with fresh medium was lower than that of the negative control sample with assay buffer (Fig. 6a), showing that the culture medium which contains phenol red in the reaction mix quenched the fluorescence signal^40,41^. This quenching effect could have possibly caused an observational error in evaluating the concentration efficiency of ISAAC by mapping 5-FAM fluorescence signal intensity. Additional calibration study with purified standard MMP-9 samples in fresh culture medium at various concentrations will be necessary to provide a more accurate quantitative evaluation of unknown MMP-9 level in biological samples.

In this study, the ISAAC platform was able to provide a semi-quantitative measurement of MMP-9 level from the standard sample as well as from the breast cancer cell culture supernatant while reducing reaction time by ~2 orders of magnitude compared to the diffusion-based enzymatic assays under static incubation. To further increase sensitivity for the detection of MMP-9 samples even at lower concentrations, either assay time can be extended to turn over more reaction product, or assay temperature (e.g., 37 °C as the optimum working temperature for MMP-9) can be increased. However, water evaporation and bubble formation inside the microchannel have to be considered since the total reaction solution volume inside the straight channel is only ~34 nL. In addition, ISAAC can initiate different preconcentration factors due to its nonlinear behaviour. Since the final fluorescence signal intensity is composed of the signal intensity of reaction product from the substrate turnover by the concentrated enzyme, as well as that of the concentrated reaction product itself, an exact quantification of the enzyme concentration remains challenging. One potential route to overcome this limitation could be by establishing calibration data of fluorescence signal intensity with a set of different MMP-9 sample concentrations, as shown in Fig. 5b, and use these data for the determination of unknown MMP-9 sample concentration. At the current stage, ISAAC could be considered as a prescreening tool, which provides rapid qualitative and semi-quantitative diagnosis in a high-throughput manner for enzyme samples with slow reaction kinetics.

## Conclusions

The goal of this study was to introduce ISAAC - an integrated assay platform based on the standard multiwell plate format with microfluidic preconcentrators - that enables high detection speed and improved detection sensitivity without adding significant operational complexities in comparison with conventional assay methods. ISAAC is constructed on one of the most widespread and disposable assay platforms - the ubiquitous multiwell plate, which offers the integrated system of ISSAC several inherent advantages such as high-throughput, device familiarity and high acceptance among users in biomedical research and clinical laboratories. To achieve this goal, we have developed a PDMS concentrator chip that can easily be multiplexed and interfaced with a standard multiwell plate. Based on the direct printing of ion-permselective polymer PEDOT:PSS onto the PDMS channel, our fabrication strategy allowed building a concentrator chip without modifying the underlying substrate and improved both repeatability and reproducibility of the device. Furthermore, this method facilitated direct interfacing of the PDMS concentrator chip with a spatially confined substrate such as a multiwell plate, which is a standard platform in high-throughput screening. Within 8 min. of assay time, our 12-well plate based concentrator platform ISAAC-12 could detect and differentiate MMP-9 with a concentration of 0, 3, 30 and 300 ng/mL, while shortening the detection time by at least ~10 hours when compared to the standard assay under static incubation. When detecting MMP-9 level from MDA-MB-231 cell culture supernatant, ISAAC enabled rapid detection (8 min. in ISAAC-12 versus 104 hours and more in a microchannel with static incubation) and increased sensitivity with a detection limit 10-times lower than that of standard incubation methods either in a microchannel or a multiwell plate. To the best of our knowledge, this study is the first attempt to integrate microfluidic preconcentrator chips into a multiwell plate to accelerate enzymatic assay without introducing additional analytical assay steps or reaction systems.

In sum, the technical novelty of the proposed work lies in the facile and straightforward way of interfacing a microfluidic device with the widely accepted high-throughput multiwell plate platform, a *de facto* standard format in laboratories, with minimal fabrication and operational complexities. As a result, increased acceptance of microfluidics in high-throughput drug screening and clinical diagnostics by lab scientists is anticipated. ISAAC can be an efficient microfluidic platform to provide rapid multiplexed enzymatic assays by enhancing free solution-based or surface binding-based assays running in a multiwell plate in terms of the detection sensitivity and speed. Its unique fabrication and assembly method allows scalability to accommodate a higher number of wells. We envision the proposed platform ISAAC as the next generation high-througput assay platform - a combination of one of the most widespread lab assay platform and the advanced microfluidics technology, that will  provide rapid detection of limited samples with high sensitivity.

## Materials and Methods

### Simulation Model for Preconcentration

To observe the preconcentration phenomenon more microscopically, we also considered a simulation model. In this section, we provide a brief description of the governing equations, simulation parameters, and boundary conditions. The simulation was carried out using an in-house code, which solved the system of Poisson-Nernst-Planck and Navier-Stokes equations on a two-dimension domain. The finite volume method was used to discretize the equations. Nonlinearity of the equations was treated directly by using the Newton's method, and discretized linear systems were solved using GMRES method. The equations were solved iteratively until convergence reached for all variables. The details about the simulation method finite volume method for discretization and Newton method for the nonlinear equations were presented in^33,35^. In our device, ion transport, electric fields, and fluid motions are governed by Nernst-Planck equations (2, 3), Poisson’s equation (4), and Navier-Stokes equations (5, 6), respectively.

2 $$\frac{\partial {{\boldsymbol{C}}}_{\pm }}{\partial {\boldsymbol{t}}}=-\,{\nabla }\cdot {\overrightarrow{{\boldsymbol{J}}}}_{\pm }$$

3 $${\overrightarrow{{\boldsymbol{J}}}}_{\pm }=-\,{\boldsymbol{F}}{{\boldsymbol{z}}}_{\pm }{{\boldsymbol{D}}}_{\pm }\nabla {{\boldsymbol{C}}}_{\pm }-\frac{{{\boldsymbol{F}}}^{2}}{{\boldsymbol{RT}}}{{\boldsymbol{z}}}_{\pm }^{2}{{\boldsymbol{C}}}_{\pm }{{\boldsymbol{D}}}_{\pm }\nabla {\boldsymbol{\phi }}+{\boldsymbol{F}}{{\boldsymbol{z}}}_{\pm }{{\boldsymbol{C}}}_{\pm }\overrightarrow{{\boldsymbol{u}}}$$

4 $${\boldsymbol{F}}({{\boldsymbol{z}}}_{+}{{\boldsymbol{C}}}_{+}+{{\boldsymbol{z}}}_{-}{{\boldsymbol{C}}}_{-})={{\boldsymbol{\rho }}}_{{\boldsymbol{e}}}=-\,\nabla \cdot ({\boldsymbol{\varepsilon }}\nabla {\boldsymbol{\phi }})$$

5 $${\boldsymbol{\rho }}(\frac{\partial \overrightarrow{{\boldsymbol{u}}}}{\partial {\boldsymbol{t}}}+\overrightarrow{{\boldsymbol{u}}}\cdot \nabla \overrightarrow{{\boldsymbol{u}}})=-\,\nabla {\boldsymbol{P}}+\,{\boldsymbol{\mu }}{\nabla }^{2}\overrightarrow{{\boldsymbol{u}}}-{{\boldsymbol{\rho }}}_{{\boldsymbol{e}}}\nabla {\boldsymbol{\phi }}$$

6 $$\nabla \cdot \overrightarrow{{\boldsymbol{u}}}=0$$

where *C*_i_, *J*_*i*_, *D*_*i*_, *z*_i_ denote the ion concentration, ion flux, ion diffusivity, and ion valence with the subscript *i* for the cation (+) or the anion (−). Time *t*, electric potential *φ*, fluid velocity *u*, and space charge *ρ*_e_ are also defined in the equations. Other constants, parameters include the Faraday’s constant *F*, gas constant *R*, electric permittivity *ε*, temperature *T*, fluid dynamic viscosity *μ*, and fluid density *ρ*.

The computation domain with a 1 cm-long region surrounding the ion-selective membrane is simulated. The region is not covering the whole microchannel but large enough to capture preconcentration phenomenon. In order to close up the system of governing equations (2–6), boundary conditions are needed. In the current modeling, the boundary conditions are designed from physical properties at the boundaries: (i) at inlet and outlet, since the boundaries are in a long distance from the membrane, ions concentration (NaCl used as electrolyte) is assumed to be the same as concentration at inlet and outlet reservoirs, 10 mM; a voltage drop set between the boundaries to remain the electric field of 3.6 V/mm (corresponding to the case of 50 V applied voltage in the experiment). (ii) in the membrane domain, as the membrane is impermeable to negative ions, a fixed negative charge is assumed distributing uniformly in the membrane area. (iii) on the PDMS and polystyrene surfaces, ions are impermeable, thus, zero-flux condition is applied to both cation and anion, i.e., ${{\boldsymbol{J}}}_{+}={{\boldsymbol{J}}}_{-}=0$, as electric field acting tangentially to the surfaces, electroosmosis velocity is applied, ${{\boldsymbol{u}}}_{{\boldsymbol{osmosis}}}=-\,\frac{{\boldsymbol{\varepsilon }}{\boldsymbol{\zeta }}}{{\boldsymbol{\mu }}}{\boldsymbol{E}}$, with ${\boldsymbol{\zeta }}$ is Zeta potential of polystyrene and PDMS substrates which are −60 mV and −40 mV, respectively.

### Device fabrication and operation

The microchannels (~17 μm high, 200 μm wide, and 1 cm long) with inlet and outlet reservoirs (4 mm in diameter) were made in PDMS using methods described elsewhere^42^. Following the previously developed protocol^32^, a ~6 μm thick high-conductivity grade polymer, PEDOT:PSS with an electrical conductivity of σ = ~150 S/cm and a zeta potential of ζ = −66.7±0.9 mV was directly printed on the centre of a PDMS microchannel with a prepatterned circle. During printing of PEDOT:PSS solution on 12 devices in a row, as in this study, the polymer solution often leaked into the side channels, resulting in different membrane edge shapes facing the sample solution and different membrane thicknesses that ultimately led to a variation in the preconcentration results. In order to confine the printed PEDOT:PSS solution inside the circular pattern, and thereby reducing such variability, we integrated three micropillar arrays on each side of the circular pattern next to the side channels. A droplet of PEDOT:PSS suspension spread inside the circular pattern with a diameter of 400 μm and remained confined without overflowing the edges. After printing, the ion-permselective conductive polymer layer was dried at room temperature for 10 min. Finally, the PDMS microfluidic concentrator was sealed against the bottom surface of each well by reversible bonding. The devices were stored at room temperature and under vacuum to ensure spontaneous filling of aqueous solutions, thereby eliminating the need for any further plasma treatment^43^.

To run assays in ISAAC-12, an electrode array consisting of 12 pairs of metal electrodes was simply inserted into each reservoir of the concentrator chips after the microchannels were filled with a reaction mix containing the target analyte. The external DC voltage (typically 10–50 V) was applied across 12 chips in the microplate through a PCB board to initiate electrokinetic concentration from a single voltage supply.

### Quantification of preconcentration performance with nucleic acids

To quantify the preconcentration efficiency of the new ISAAC device in comparison with our previous concentrator devices which were bonded onto a glass substrate, 50 μL of cyanine 5 (Cy5)-labelled DNA target (5′ CAA CCG ATG CCA CAT CAT TAG CTA C-Cy5 3′) with an initial concentration (C_0_) ranging from 0.1 nM to 10 nM in 0.1X phosphate-buffered saline (PBS) was loaded in the anodic reservoir of a concentrator chip. 100 μL of 0.1X PBS at pH 7.1 was loaded into the cathodic reservoir. A constant DC voltage of 50 V was applied across the microfluidic channel to initiate electrokinetic preconcentration based on ICP. To quantify the concentration increase, fluorescence signal intensity was measured and compared to the signal intensity of known reference DNA concentrations. All experiments were run in triplicate. Preconcentration performance data of the same target from previously reported devices, in which PEDOT:PSS was directly printed onto a PDMS chip^31^ and then reversibly bonded onto a glass substrate, were used as references.

### Application in matrix metalloproteinase (MMP)-9 detection

MMP-9 belongs to the metzincin family of mostly extracellularly operating proteases, and is a type IV collagenase and 92-kDa protein that is encoded by a gene located on chromosome 20 (20q13.12) in the human genome^44^. Studies over the last decades have proven the significant correlation between MMP-9 expression level in cells as well as extracellular vesicle exosomes and tumour metastasis^45–49^, which make it an important biomarker in liquid biopsy. A commercially available assay kit - SensoLyte^®^ 520 MMP-9 Assay Kit *Fluorimetric* (AnaSpec Inc.) was purchased and used to prepare the reaction mix for MMP-9 detection from both standard MMP-9 samples as well as MMP-9 samples collected from MDA-MB-231 cell culture supernatants. The presence of MMP-9 can cleave off the fluorophore 5-FAM from the FRET peptide substrates labelled with a fluorophore 5-FAM and its quencher QXL520^™^ in the reaction reagent. The recovered 5-FAM can provide continuous measurement of the enzyme activities.

### Preparation of MMP-9 samples

Purified human MMP-9 (Recombinant, Catalytic Domain) (AnaSpec Inc.) was used as a standard sample to demonstrate the capability of ISAAC in increasing enzymatic reaction rate. The MMP-9 samples were prepared at four different concentrations from 0 (negative control: NC), 3, 30, to 300 ng/mL with assay buffer provided with the kit. Human breast cancer cell line MDA-MB-231 was purchased from Sigma (MilliporeSigma, St. Louis, MO) and maintained in Dulbecco’s Modified Medium (DMEM)-high glucose supplemented with 10% FBS and penicillin (100 U/mL)/streptomycin (100 μg/mL) according to the supplier’s recommendation. Cells were plated in a culture flask and cultured for 5 days in a 5% CO_2_ incubator at 37 °C, with a medium change at 48 h prior to the collection of culture supernatant. The number of cells was 4.2 × 10^7^/mL at the time of collection. Cell culture medium was centrifuged at 1000 × g and 4 °C for 10 min to remove cell debris. The supernatant was aliquoted in 2 mL cryo tubes and stored at −80 °C. Two positive samples were prepared to measure MMP-9 expression level: 1X MMP-9 cell culture supernatant sample (without dilution) and 0.1X MMP-9 cell culture supernatant sample (10-fold dilution with assay buffer). Three negative samples were prepared to eliminate possible assay error: negative control (assay buffer); negative control+1X medium (fresh culture medium); negative control+ 0.1X medium (10-fold dilution of fresh medium with assay buffer).

According to the manual instruction, 40 μL of the reaction mix including 20 μL reaction substrate and 20 μL MMP-9 sample was prepared and loaded into each concentrator chip of the ISAAC-12 platform. An external DC voltage of 10 V was applied across all the microchannels to start ICP and the fluorescence signal intensity of reaction product 5-FAM was monitored in each well over 8 min. For comparison, standard assays under static incubation of the reaction mix were also performed both in an array of microchannels without ICP as well as in a 384-well plate at room temperature. The fluorescence signal intensity of reaction product in the microchannels and in the 384-well plate was measured with an EMCCD camera (ANDOR, iXon EMCCD 897) and Varioskan® Flash plate reader (Thermo Scientific), respectively. For data analysis, Fiji and OriginPro® (OriginLab) were used.

### Statistical analysis

Kruskal-Wallis ANOVA test was used to evaluate the difference among groups using OriginPro^®^ (OriginLab). Significant differences were defined as p < 0.05 (significance level α = 0.05).

### Data availability

The datasets generated and/or analyzed during the current study are available from the corresponding author on reasonable request.

## Electronic supplementary material

Supplementary Information
