# Author Correction: A Multiwell-Based Detection Platform with Integrated PDMS Concentrators for Rapid Multiplexed Enzymatic Assays

**DOI:** 10.1038/s41598-018-33002-z

**Published:** 2018-10-02

**Authors:** Xi Wei, Vu Q. Do, Sang V. Pham, Diogo Martins, Yong-Ak Song

**Affiliations:** 1 Division of Engineering, New York University Abu Dhabi, Abu Dhabi, United Arab Emirates; 2 Department of Chemical and Biomolecular Engineering, Tandon School of Engineering, New York University, Brooklyn, United States; 3 School of Transportation Engineering, Hanoi University of Science and Technology, No1 DaiCoViet, Hanoi, Vietnam; 4 NOVA Medical School, Faculdade de Ciências Médicas, Universidade Nova de Lisboa, Lisboa, Portugal

Correction to: *Scientific Reports* 10.1038/s41598-018-29065-7, published online 17 July 2018

The Acknowledgements section in this Article is incomplete.

“We gratefully acknowledge the financial support from New York University Abu Dhabi (NYUAD) through the NYUAD Research Enhancement Fund as well as the ADEK Award for Research Excellence 2015 (Award number: AARE-073). The device fabrication was conducted in the microfabrication core facility of NYUAD.”

should read:

“We gratefully acknowledge the financial support from New York University Abu Dhabi (NYUAD) through the NYUAD Research Enhancement Fund as well as the ADEK Award for Research Excellence 2015 (Award number: AARE-073). The device fabrication was conducted in the microfabrication core facility of NYUAD.

V.S. Pham and Q.V. Do are funded by Vietnam National Foundation for Science and Technology Development (NAFOSTED) under grant number 107.03-2016.11.”

