# Supplementary material for: A Multiwell-Based Detection Platform with Integrated PDMS Concentrators for Rapid Multiplexed Enzymatic Assays
Source: Sci Rep. 2018 Jul 17;8:10772. doi: 10.1038/s41598-018-29065-7 (PMC6050343; doi:10.1038/s41598-018-29065-7)
Supplement: Supplementary file 1 — Supplementary Information [file 41598_2018_29065_MOESM1_ESM.docx]

Supplementary Information

A Multiwell-Based Detection Platform with Integrated PDMS Concentrators for Rapid Multiplexed Enzymatic Assays

Xi Wei^a,b^, Vu Q. Do^c^, Sang V. Pham^c^, Diogo Martins^d^ &Yong-Ak Song ^a,b*^

^a^Division of Engineering, New York University Abu Dhabi, Abu Dhabi, United Arab Emirates

^b^Department of Chemical and Biomolecular Engineering, Tandon School of Engineering, New York University, Brooklyn, United States

^c^School of Transportation Engineering, Hanoi University of Science and Technology, No1 DaiCoViet, Hanoi, Vietnam

^d^NOVA Medical School, Faculdade de Ciências Médicas, Universidade Nova de Lisboa, Lisboa, Portugal.

* To whom correspondence should be addressed, E-mail: [rafael.song@nyu.edu](mailto:rafael.song@nyu.edu); Phone: +971-2-628-4781; Fax: +971-2-659-0794

Table of Content

Figure S1-S4 S2-S3

Table S1 s4


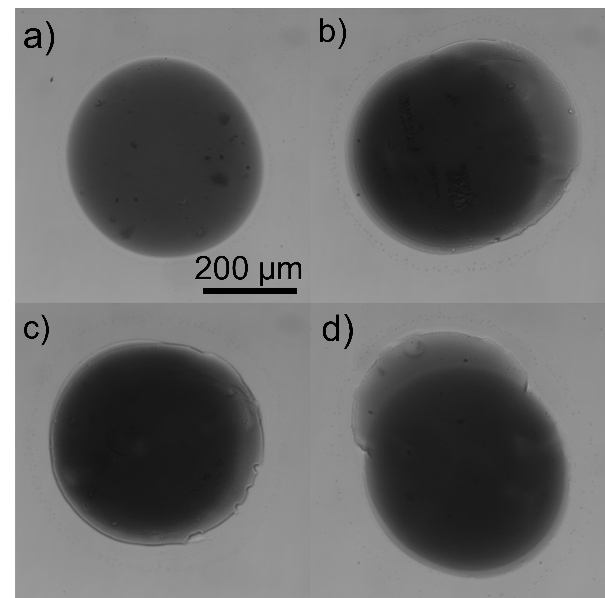


**Figure S1.** Optical images of the typical PEDOT:PSS membrane with ~ 6 μm thickness on a glass substrate by using layer-by-layer printing technique. a) An example of a PEDOT:PSS membrane which can be used for concentration experiments. b-d). Examples of failed membranes due mismatched layers as results of either pin position misalignment between printing steps (b), or overflow of the second membrane layer caused by increased dispensing volume associated with viscosity change (c and d).


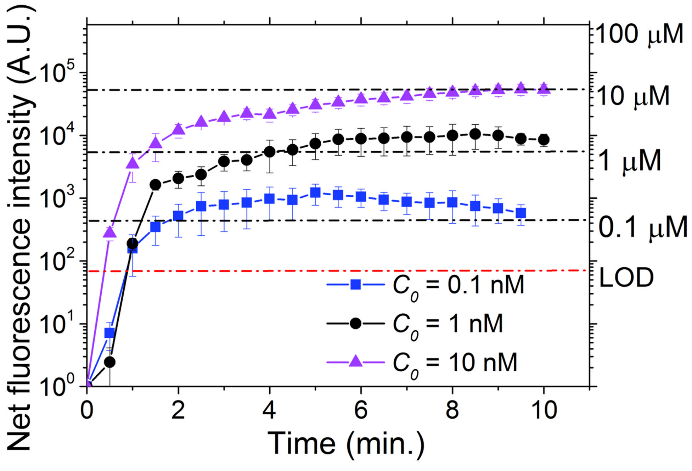


**Figure S2.** Quantitative characterization of electrokinetic concentration in ISAAC-12 by measuring the electrokinetically concentrated Cy5 tagged DNA with an initial concentration *C_0_* from 0.1 nM to 10 nM in 0.1X PBS at DC 50 V. The preconcentration factor obtained was about 10^3^ within 10 min.

**Figure S3.** Comparison study of MMP-9 assay by 9.5 h incubation with positive control (300 ng/mL) and its negative control in a) the microchannel without electrokinetic enrichment and b) the 384-well plate. After 9.5 h incubation, positive signal was 1.5 and 3.6-fold higher than the negative control in the microchannel and microwell, respectively. The difference in signal level between the two devices was due to i) the fluorescence detector sensitivity difference between microscope and plate reader; ii) the difference in reaction volume used for quantification.

**

**

**Figure S4.** The standard calibration curve based on the 18 h incubation assay of MMP-9 standard samples with concentrations at 0, 30, 300 and 3000 ng/mL in the 384-well plate of. All results were calculated from triplicate assay data. The concentration of 1X MMP-9 supernatant sample from MDA-MB-231 breast cancer cell culture was calculated as 160.2 ng/mL based on fluorescence readings (y value of the cyan color dot = 2.06) by the plate reader and the linear fitting equation displayed next to the plotted curve. The concentration of 10X MMP-9 supernatant sample was determined as ~16 ng/mL since it is the 10-fold dilution of 1X MMP-9 supernatant sample.

The intra- and inter-assay coefficient of variation (cv) calculation of MMP-9 assay in ISSAC-12.

The CV was calculated as the ratio of the sample standard deviation (SD) to the sample mean. The intra (within-experiment) -assay CV was determined by measurements from 3 triplicates for 4 MMP-9 samples with concentration at 0, 3, 30 and 300 ng/mL. Inter (between-experiments) -assay CV was determined by measurements from 4 MMP-9 samples with concentration at 0, 3, 30 and 300 ng/mL in 3 independent experiments. The overall intra- and inter-assay CV of ISAAC-12 was assessed by calculating the mean of CV from each group.

**Table S1.** The intra- and inter-assay coefficient of variation (CV) calculation of MMP-9 assay in ISAAC-12.

| MMP-9 sample concentration (ng/mL) | CV % | |
| --- | --- | --- |
|  | Intra-assay | Inter-assay |
| 0 | 8.61 | 6.93 |
| 3 | 10.32 | 10.91 |
| 30 | 11.66 | 9.95 |
| 300 | 8.59 | 10.03 |
| Overall performance of ISAAC-12 | 9.8 | 9.45 |
